# Vitamin D Receptor Gene Polymorphism: An Important Predictor of Arthritis Development

**DOI:** 10.1155/2019/8326246

**Published:** 2019-03-18

**Authors:** Maryam Mukhtar, Nadeem Sheikh, Saira Kainat Suqaina, Andleeb Batool, Naz Fatima, Rabia Mehmood, Sabeen Nazir

**Affiliations:** ^1^Cell and Molecular Biology Lab, Department of Zoology, University of the Punjab, Quaid-i-Azam Campus, Lahore 54590, Pakistan; ^2^Department of Zoology, Government College University, Lahore 54000, Pakistan

## Abstract

Vitamin D is an anti-inflammatory molecule and has a role in prevention of arthritis development. Biologically active form 1, 25(OH)_2_D3 of vitamin D can only exert its action after binding its definite vitamin D receptor encoded by VDR gene. VDR gene polymorphism leads to dysfunctioning of 1, 25(OH)_2_D_3_ ultimately disease onset. The purpose of current study was to evaluate the effect of vitamin D level and VDR gene polymorphism on rheumatoid arthritis and osteoarthritis. Blood samples were collected from case and control after taking written consent. Serum was separated and vitamin D level as determined from each sample by ELISA. DNA was extracted from each blood sample and amplified by using gene specific primers. Genotyping was performed by Sangers sequencing and PCR-RFLP technique. It was found that vitamin D level was not significantly different among patients and controls. The rs10735810, rs1544410, rs7975232, and rs731236 were associated with the onset of arthritis at both allelic and genotypic level (p < 0.01). Nucleotide change on rs10735810 site leads to change of tryptophan with arginine. The frequencies of haplotype CGAT, CGGA, CGGT, CTAA, CTAT, TGAA, TGAT, TGGA, and TTGA were higher in patients and act as risk factors of RA onset, whereas haplotypes CGAT, CGAT, CGGT, CTGA, TGAT, TGGA, TTAA, and TTGA were associated with OA onset. In conclusion, serum vitamin D level may be normal among arthritis patients but polymorphism on VDR gene restricts vitamin D to perform its anti-inflammatory function by altering the 1, 25(OH)2 D3 binding sites.

## 1. Introduction

1, 25 dihydroxy vitamin D3 is pleiotropic steroid formed in skin by sunlight exposure from its precursor [1]. It acts as an immunoregulator by activating human lymphocytes that express its steroid receptor, vitamin D receptor (VDR), and inhibit antibody production and interleukin-2 along with suppression of cytotoxic lymphocytes and their proliferation [2]. Vitamin D3 is known to play a key role in calcium homeostasis that is in metabolism of minerals by bonding to its receptor (VDR). It can regulate bone turnover by stimulating both osteoclastic and osteoblastic linage cells and can also stimulate bone reabsorption and formation [3].

Ultimately, all these effects of vitamin D influence rheumatoid arthritis (RA) as well as osteoarthritis (OA). VDR is majorly located on immune cells including CD4+ T cells, CD8+ T cells, and antigen-presenting cells [2]. After the entrance of vitamin D2 or D3, it forms complex with the vitamin D binding protein (VDBP) and then makes its way to the liver. In liver, hydroxylation of vitamin D occurs at position 25 to form 25-hydroxyvitamin D (25[OH]D). 25-hydroxyvitamin D (25[OH]D) is then transported to the kidney where it again undergoes hydroxylation by the action of 1-hydroxylase enzyme and forms 1,25 dihydroxy vitamin D (1,25[OH]2D) (active hormonal form) and then bounds to the VDBP to reach the target cell. It binds to cytoplasm VDR and forms complex after entering the cell. This complex enters in nucleus where it heterodimerizes with retinoic acid X receptor and enhances vitamin D-dependent genes transcription crucial in bone and calcium metabolism [4].

The gene (VDR) encoded for VDR is located on chromosome 12 employs a critical effect on the immune system in general and the Th1 cells in particular [5]. Numerous variations on VDR have been recognized in patients of multiple disorders. Majority of them cause VDR to be unable to bind to 1, 25-OH-D. Over 63 polymorphisms on VDR gene were reported in different populations out of which rs10735810, rs1544410, rs7975232, and rs731236 were extensively studied [6], whereas very few studies investigate association of VDR gene polymorphism with OA.

In Pakistan, 70% of marriages are cousin marriage which is the major reason of transferring genetic disorders to next generation. Unfortunately, most of the times we are relying on the information provided in the foreign research which may not fit because of different genetic background. There is lack of baseline data that is required with reference to our population. Therefore, the present study was conducted with the aim to investigate the linkage of vitamin D level and VDR gene polymorphism with disease onset in Pakistani population.

## 2. Materials and Methods

### 2.1. Subjects

The current study was ethically approved by the Punjab University Advanced Studies and Research Board, Lahore, Pakistan. This case-control study was carried on RA and OA patients recruited from the rheumatology and orthopedic center of Public and Semigovernment Hospitals of Punjab, Pakistan. Written consent was obtained from patients of the studied subjects as well as from controls. All participants were clinically diagnosed with RA and OA by a physician according to WHO criteria. The inclusion criteria for RA patients include patients with positive RF factor and who are eligible for Rituximab therapy according to UK NICE guidelines; 2010 ACR/EULAR Rheumatoid Arthritis classification criteria for a diagnosis of RA; participants of 18 years of age or over must be capable of giving informed consent. On the other hand, the inclusion criteria for OA include patients that are overweight, previous knee injury or surgery, and knee pain during the past year. All self-diagnosed patients who will have incomplete tests reports will be excluded from the study. A total of 300 RA cases, 316 OA cases, and 412 controls were included in the study. The clinical characteristics of the participants were presented in Table 1. All control subjects were healthy and had a negative family history of arthritis.

### 2.2. ELISA

Blood sample (2cc) from each participant was collected in gel-coated tube and serum was extracted by spinning tubes at 5000 rpm for 15 minutes. ELISA was performed according to manufacturer (CALBIOTECH) instruction for determination of serum vitamin D by using 25 (OH) vitamin D ELISA Kit (Cat No. VD220B). The minimum detectable dose (MDD) of kit used was 0.67ng/ml.

### 2.3. DNA Isolation and SNPs Selection

Blood samples (3cc) from each case and control were collected in EDTA-coated tubes and DNA was extracted by the modified organic extraction method [7]. For further genetic analysis, extracted DNA from each sample was stored at -20^*ο*^C (Haier). Nanodrop was performed for DNA quantification by the nanodrop (Thermo 2000) method. Four polymorphic sites rs10735810, rs1544410, rs7975232, and rs731236 were selected by using the SNP Browser software 4.0 (Applied Biosystems) and Hap Map database (http://hapmap.ncbi.nlm.nih.gov/).

### 2.4. Polymerase Chain Reaction

The targeted DNA fragment was amplified for Polymerase Chain Reaction (PCR) in a 25*µ*l reaction mixture by using following primers: for rs10735810 polymorphic site “Forward primer (F.P): AGCTGGCCCTGGCACTGACTCTGGCTCT and Reverse Primer (R.P): ATGGAAACACCTTGCTTCTTCTCCCTC”, for rs7975232 and rs731236 polymorphic site “F.P: CAGAGCATGGACAGGGAGCAA and R.P: GCAACTCCTCATGGCTGAGGTCTC”, and for rs1544410 polymorphic site “F.P: CAACCAAGACTACAAGTACCGCGTCAGTGA and R.P: AACCAGCGGGAAGAGGTCAAGGG.” For all primers, the PCR reaction included 35 cycles, which consist of initial denaturation at 95°C for 5:00 mins, denaturation at 95°C for 45s, annealing at 68°C (rs10735810), 65°C (rs7975232 and rs731236), and 55.9°C(rs1544410) for 45s, and extension at 72°C for 30s followed by final denaturation at 72°C for 10 mins.

### 2.5. VDR Genotyping

VDR genotyping for rs10735810, rs1544410, rs7975232, and rs731236 was performed by direct sequencing method and Restriction Fragment Length Polymorphism (PCR-RFLP). The sequences were visualized on Bio Edit software and mutations were determined and were confirmed by NCBI BLAST. For PCR-RFLP, according to the manufacturer's instructions, the PCR products were digested using restriction enzymes: FokI (rs10735810) (Fermentas, Germany), ApaI (rs731236), TaqI (rs7975232), and BsmI (rs1544410) (Vivantis). Briefly, a reaction mixture of 25 *µ*l containing 10 *µ*l of each PCR product was mixed with 2 *µ*l of Tango buffer, 1 *µ*l of restriction enzyme, and 12 *µ*l of DEPC water. The tubes were incubated at 55°C for 5h (FokI), 37°C for 16h (ApaI), 65°C for 3h (TaqI), and 37°C for 5h (BsmI), followed by thermal inactivation of restriction enzymes at 80°C (FokI and TaqI) and 65°C (ApaI and BsmI), for 20 mins. Digested samples were run on 2% agarose gel and visualized on the gel documentation system (BioDoc-It Imaging System).

### 2.6. Statistical Analysis

Vitamin D significance level was calculated by applying ANOVA. All genetic data of the controls passed the Hardy-Weinberg Equilibrium (p> 0.05). The Chi-Square test was used to determine allelic and genotypic frequencies. Regression (dominant and recessive) analysis was performed. Linkage disequilibrium and haplotype were calculated to study their association with RA and OA by SHEsis (http://analysis.bio-x.cn/myAnalysis.php). The change in amino acid sequences was determined by aligning sequences in Mega 6 software.

## 3. Results

It was observed that overweight and obesity were significantly associated with onset of RA as well as OA (p < 0.01). Both positive paternal and maternal family history of arthritis were significant risk factors of disease development.

**Figure 1:**
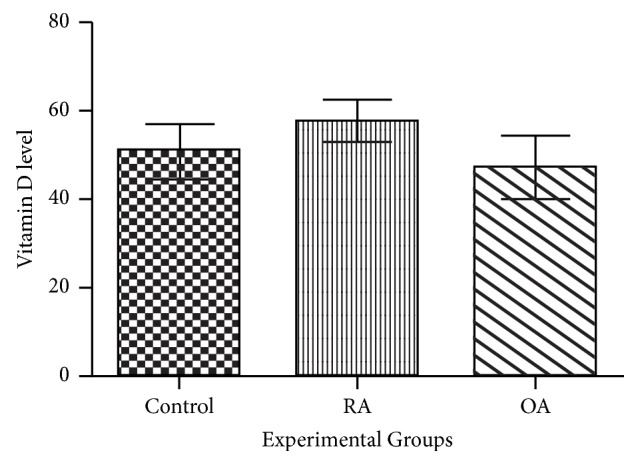
Comparison of serum vitamin D level among RA, OA, and controls.

As a result of ELISA, it was found that in serum 25(OH)_2_D_3_ was sufficient among RA, OA, and controls (30 ng/ml – 100ng/ml) and there was no significant difference in 25(OH)_2_D_3_ level among the studied groups (Figure 1).

For genetic analysis, Hardy-Weinberg Equilibrium (HWE) was applied on all genetic data and was observed that all SNPs followed HWE (p > 1.00).

Single-site analysis was performed and was presented in Table 2. It was observed that, on rs10735810 polymorphic site on exon 2, allele ‘C' acts as risk allele and is significantly associated with the onset of RA as well as OA (p = 0.016). The odd ratio and coefficients interval calculated were 1.42 and 0.82~2.44 in RA, whereas they were 1.11 and 0.64 ~1.89 in OA individuals. On rs7975232 and rs731236 polymorphic sites, allele ‘G' replaced allele ‘T' and allele ‘A' replaced allele ‘T' and acted as susceptible risk alleles in both arthritis groups (RA {O.R=2.43, %95 CI= 1.74~3.38} OA {O.R=1.40; 95% CI=1.00~1.96}) whereas, on rs1544410 polymorphic site in RA patients, allele ‘A' replaced allele ‘G' but was not significantly associated with disease onset. On the other hand, rs1544410 was found to be significantly associated with disease onset at allelic level in OA subjects.

As a result of genetics analysis shown in Table 3, it was observed that in cases frequency of ‘CC' and ‘CT' was more prevalent as compared to controls on rs10735810 polymorphic site. On rs7975232, ‘GG' and ‘GT' genotypes were significantly associated with RA onset and not significantly associated with the development of OA in patients. The frequency of genotypes ‘AA' and ‘AT' was higher in all cases as compared to controls on rs1544410 polymorphic site whereas genotype ‘TT' was higher in controls. rs1544410 was found to be significantly associated with the onset of RA and OA at genotype level as genotypes ‘AA' and ‘AG' frequency significantly differed among patients and control subjects.

As a result of regression analysis, it was found that all the SNPs were significantly associated with the onset of RA as well as OA (Table 4).

Haplotype analysis was presented in Table 5 and it was observed that, in RA and OA subjects, haplotype TTGT frequency is higher in controls and protective against disease onset, whereas haplotype CGAT, CGGA, CGGT, CTAA, CTAT, TGAA, TGAT, TGGA, and TTGA were significantly associated with the onset of RA. On the other hand, in OA subjects, haplotype CGAT, CGAT, CGGT, CTGA, TGAT, TGGA, TTAA, and TTGA were associated with disease onset.

**Figure 2:**
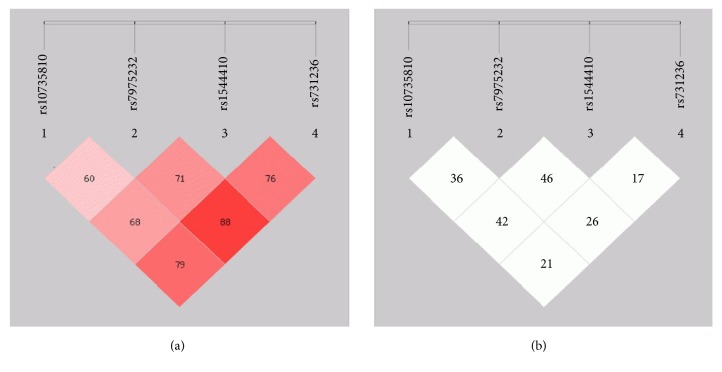
Location and map of linkage disequilibrium (LD) in SNPs at* VDR* gene in RA are presented. The SNPs numbers are indicated at the top of haploview. (a) LD = D^/^ (b) LD coefficient.

**Figure 3:**
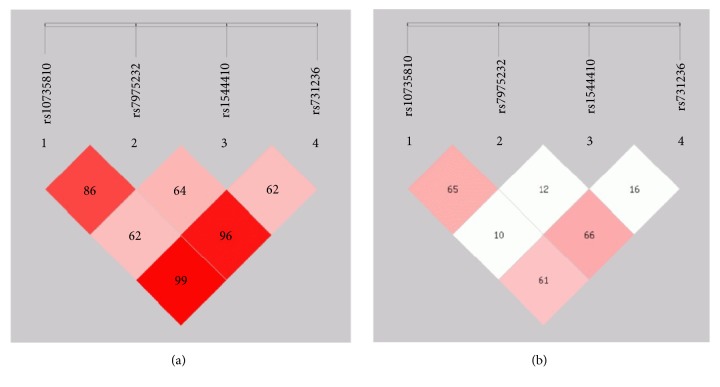
Location and map of linkage disequilibrium (LD) in SNPs at* VDR* gene in OA are presented. The SNPs numbers are indicated at the top of haploview. (a) LD = D^/^ (b) LD coefficient.

As a result of linkage disequilibrium (LD) analysis (Figures 2(a), 2(b), 3(a), and 3(b)), in RA patients vs. controls, it was observed that rs731236 along with rs1544410 (D'=0.795; r2=0.211) followed by rs7975232 along with rs1544410 (D'=0.688, r2=0.426) and then rs10735810 in combination with rs7975232 were linked with RA onset (D' = 0.604; r2=0.364), whereas variants on rs731236, rs1544410, and rs7975232 increased chances on RA development as compared to that of controls (D'=0.889, r2=0.264) followed by rs1544410, rs10735180, and rs7975232 (D'=0.717, r2=0.416). On the other hand, in OA subjects and controls, rs10735810 and rs7975232 in combination were highly associated with disease onset (D'=0.866, r2=0.654). rs7975232 along with rs1544410 (D'=0.629, r2=0.103) and rs731236 along with rs1544410 (D'=0.995, r2=0.618) were also associated with OA onset. rs731236, rs1544410, and rs7975232 if passed together to next generation raised risk of OA development as compared to that of controls (D'=0.967, r2=0.670) followed by rs1544410, rs10735180, and rs7975232 (D'=0.650, r2=0.126). If all the polymorphic sites will be forwarded to next generation in both RA and OA cases, then the chances of disease development will be highly increased (D'=0.765, r2=0.176; D'=0.623, r2=0.162) respectively.

Amino acid alignment on Mega 6 software showed that change in nucleotide on rs10735180 polymorphic site ultimately leads to the change of tryptophan with arginine. The variation on rs731236, rs1544410, and rs7975232 polymorphic site will not lead to the change of amino acid sequence.

## 4. Discussion

Vitamin D has been verified as a vital factor in the onset of autoimmune disorders [8]. In Pakistani population, cousin marriages lead to the transmission of diseases more often to next generation ultimately increasing the risk rate of genetic disorders onset. However, the current study was conducted to determine the vitamin D level in RA as well OA patients and to determine the susceptibility of VDR gene polymorphism with the onset of arthritis.

It was demonstrated that high BMI has been significantly associated with arthritis onset. Similarly, a study reported that obesity is highly prevalent among arthritis persons [9]. Another study reported that it is a modifiable threat linked with disease progression, disability, total joint replacement, activity limitation, and reduced quality of life (Centers for Disease Control and Prevention) [10]. Two studies also concluded a direct significant association between RA onset probability and obesity [11, 12]. Nurse health study also reported that risk of RA increases with obesity before 55 *ο*f age (HR 1.65; 95% CI 1.34 to 2.05 [13]. In a pipeline of current findings, a study demonstrated that the risk of OA increases up to 36% with every weight gain of 50kg [14]. High BMI also influences OA severity as in comparison to normal or underweight subjects, obese people have significant knee joint degradation [15]. Precisely, OA and obesity limited mobility leads to further gaining of weight, reduced muscles strength, and ultimately progression of joint issues and disease [16].

Current study demonstrated that no significant difference was observed in serum vitamin D level in RA, OA, and control subjects. A case control study conducted on Thai population also reported no association between RA and serum vitamin D level [17]. Similarly, another study reported that serum 25 (OH) D levels were not associated with the radiographic knee OA severity and its functional assessment [18]. In contrast to current findings, frequent studies from multiple geographical regions and countries and their meta-analysis recommended significant inverse correlation between vitamin D and disease onset in RA patients [19–21]. Significant clinical benefits with respect to pain and function of vitamin D treatment in OA patients were reported [22]. Glover et al. [23] also demonstrated that the intensity of knee OA pain and function decreased in patients with adequate vitamin D level.

Current study demonstrated the strong association of VDR gene polymorphism (rs10735810, rs7975232, rs731236, and rs1544410) with the onset of RA and OA in studied population. Various studies also reported significant association of VDR gene polymorphism in Egyptian (Mansoura) (rs7975232, rs731236, and rs1544410), French (rs10735810), Canadian (North American Natives) (rs10735810), and Tunisian (rs10735810) populations [24–27]. Meanwhile, contrary to this, no significant association was reported in Korean (rs1544410, rs7975232), Spanish (rs7975232, rs731236, and rs1544410), Hungarian (rs1544410), French (rs1544410, rs7975232), Tunisian (rs7975232, rs731236, and rs1544410), Turkish (rs1544410, rs7975232, and rs10735810), Egyptian (Mansoura) (rs10735810), Indian (rs10735810), German (rs10735810, rs1544410, and rs7975232), and Egyptian (Zagazig) (rs1544410) populations [25–34]. Similarly, in Chinese population, no association was reported between OA and VDR gene polymorphism [35].

It was found that haplotype TTGT was protective against disease onset whereas haplotype CGAT, CGGA, CGGT, CTAA, CTAT, TGAA, TGAT, TGGA, and TTGA were significantly associated with the onset of RA. On the other hand, in OA subjects, haplotype CGAT, CGAT, CGGT, CTGA, TGAT, TGGA, TTAA, and TTGA were associated with disease onset.

Current study demonstrated that polymorphism on rs10735810 leads to the change of tryptophan with arginine. Mukhtar et al. [36] also reported that polymorphism at rs10735810 polymorphic site on VDR gene leads to the substitution of arginine.

Current findings revealed that among Pakistani RA and OA subject's serum vitamin D level was not significantly low but polymorphism on VDR gene did not enable vitamin D to attain its active form and act to prevent disease onset.

In conclusion, high BMI and positive maternal and paternal family history are significant factors in the onset of RA as well as OA. Moreover, vitamin D level is not significantly inadequate but VDR gene polymorphism is a significant risk factor of RA as well as OA onset in Pakistani population.

## Figures and Tables

**Table 1:** Clinical Characteristics of participitants.

Sr No.	Parameters	RA (n=300)		OA (n=316)	
		Males (n=71)	Females (n=229)	Males (n=100)	Females (n=216)
1.	Age (Years)	38.62 (35.94 ± 41.30)	38.98 (37.42 ± 40.55)	55.27 (52.88 ± 57.66)	49.19 (47.79 ± 50.58)
2.	BMI (Kg/m^2^)	24.56 (23.45 ± 25.68)	26.43 (25.70 ± 27.16)	30.40 (29.20 ± 31.61)	30.00 (29.32 ± 30.69)
3.	Age of Diagnosis (Years)	33.06 (30.55 ± 35.56)	31.56 (29.97 ± 33.15)	46.95 (44.84 ± 49.06)	42.73 (40.61 ± 44.85)
4.	Disease Duration (Years)	5.83 (4.436 ± 7.223)	7.27 (6.464 ± 8.075)	7.06 (5.690 ± 8.436)	5.64 (4.985 ± 6.295)
5.	Positive Family History (%)	85.92	80.35	69.00	68.98

BMI = Body Mass Index; n = No of individuals; RA = Rheumatoid arthritis; OA = Osteoarthritis.

**Table 2:** Allelic test of genetic variants in RA, OA and controls.

SNPs	Alleles	RA Frequency (P/C)	*X* ^*2*^-*value*	p-value	OA Frequency (P/C)	*X* ^*2*^-*value*	p-value
rs10735810	C	0.723/0.018	799.60	0.016*∗*	0.671/0.018	723.47	0.016*∗*
	T	0.277/0.982			0.329/0.982		

rs7975232	G	0.745/0.001	890.75	0.016*∗*	0.628/0.001	707.78	0.015*∗*
	T	0.255/0.999			0.372.0.999		

rs1544410	A	0.803/0.000	1000.64	>0.01	0.233/0.000	213.18	0.016*∗*
	G	0.197/1.000			0.767/1.000		

rs731236	A	0.315/0.000	299.28	0.015*∗*	0.489/0.000	511.40	0.016*∗*
	T	0.685/1.000			0.511/1.000		

P= Patients; C= Control; **∗** represents significance at the 0.01 level.

**Table 3:** Genetic test of genetic variants in RA, OA and controls.

SNPs	Genotypes	RA Frequency (P/C)	*X* ^*2*^ *-value*	p-value	OA Frequency (P/C)	*X* ^*2*^ *-value*	p-value
rs10735810	CC	0.537/0.000	554.05	0.016*∗*	0.345/0.000	667.02	0.017*∗*
	CT	0.373/0.036			0.652/0.036		
	TT	0.090/0.964			0.003/0.964		

rs7975232	GG	0.530/0.000	660.108	0.016*∗*	0.278/0.000	695.92	>0.01
	GT	0.430/0.002			0.699/0.002		
	TT	0.040/0.998			0.022/0.988		

rs1544410	AA	0.697/0.000	608.07	0.008*∗*	0.098/0.000	179.90	0.005*∗*
	AG	0.213/0.000			0.269/0.000		
	GG	0.090/1.000			0.633/1.000		

rs731236	AA	0.050/0.000	316.24	0.005*∗*	0.000/0.000	699.98	0.015*∗*
	AT	0.530/0.000			0.978/0.000		
	TT	0.420/1.000			0.022/1.000		

P= Patients; C= Control; **∗** represents significance at the 0.01 level.

**Table 4:** Regression analysis (Studied SNP's association with response Status (n=712, adjusted by Sex+Age+BMI).

SNPs	Model	Genotype	RA					OA				
			Status=Cases	Status=Control	P-value	AIC	BIC	Status= Cases	Status= Control	P-value	AIC	BIC
rs10735810	Dominant	T/T	27 (9%)	397 (96.4%)	<0.0001	321.7	344.5	1 (0.3%)	397 (96.4%)	<0.0001	132.1	159.5
		C/T-C/C	273 (91%)	15 (3.6%)				309 (99.7%)	15 (3.6%)			
	Recessive	T/T-C/T	139 (46.3%)	412 (100%)	<0.0001	611.4	634.3	204 (65.8%)	412 (100%)	<0.0001	702.8	730.3
		C/C	161 (53.7%)	0 (0%)				106 (34.2%)	0 (0%)			

rs7975232	Dominant	T/T	12 (4%)	411 (99.8%)	<0.0001	123.3	146.1	6 (1.9%)	411 (99.8%)	<0.0001	52.7	80.2
		G/T-G/G	288 (96%)	1 (0.2%)				304 (98.1%)	1 (0.2%)			
	Recessive	T/T-G/T	141 (47%)	412 (100%)	<0.0001	637	659.8	227 (73.2%)	412 (100%)	<0.0001	735.8	763.3
		G/G	159 (53%)	0 (0%)				83 (26.8%)	0 (0%)			

rs1544410	Dominant	G/G	27 (9%)	412 (100%)	<0.0001	208.8	231.7	194 (62.6%)	412 (100%)	<0.0001	562.5	590
		A/G-A/A	273 (91%)	0 (0%)				116 (37.4%)	0 (0%)			
	Recessive	G/G-A/G	91 (30.3%)	412 (100%)	<0.0001	484.6	507.5	79 (90%)	412 (100%)	<0.0001	771	798.5
		A/A	209 (69.7%)	0 (0%)				31 (10%)	0 (0%)			

rs731236	Dominant	T/T	126 (42%)	412 (100%)	<0.0001	510.6	533.4	-	-	-	-	-
		A/T-A/A	174 (58%)	0 (0%)				-	-			
	Recessive	T/T-A/T	285 (95%)	412 (100%)	<0.0001	947	969.8	-	-	-	-	- -
		A/A	15 (5%)	0 (0%)				-	-			

AIC=Akaike information criterion; BIC=Bayesian information criterion; P-value<0.01=significant.

**Table 5:** Haplotype Analysis of the *VDR *gene located on chromosome 12.

Haplotype: rs10735810; rs7975232; rs1544410; rs731236					
Haplotype	RA Frequency (P/C)	p-value	Haplotype	OA Frequency (P/C)	p-value

CTGT*∗*	0.030/0.018	0.140	CTGT	0.015/0.018	0.651
TGGT*∗*	0.021/0.001	0.001	TGGT*∗*	0.004/0.001	0.001
TTGT	0.002/0.981	0.001	TTGT*۞*	0.000/0.981	0.016
CGAT*∗*	0.301/0.000	0.016	CGAT*∗*	0.102/0.000	0.016
CGGA*∗*	0.071/0.000	0.014	CGGA*∗*	0.088/0.000	0.015
CGGT*∗*	0.041/0.000	0.009	CGGT*∗*	0.379/0.000	0.016
CTAA*∗*	0.011/0.000	0.002	CTAA	0.001/0.000	>0.01
CTAT*∗*	0.188/0.000	0.016	CTGA*∗*	0.086/0.000	0.015
TGAA*∗*	0.120/0.000	0.015	TGAT*∗*	0.011/0.000	0.002
TGAT*∗*	0.102/0.000	0.015	TGGA*∗*	0.044/0.000	0.001
TGGA*∗*	0.007/0.000	0.001	TTAA*∗*	0.118/0.000	0.001
TTGA*∗*	0.026/0.000	0.006	TTGA*∗*	0.152/0.000	0.001

*∗*Represents a significant association of Haplotypes with Arthritis onset.

۞ Represents a significant association of Haplotypes protective against Arthritis onset.

## Data Availability

The data used to support the findings of this study are available from the corresponding author upon request.
